# Extracellular Alkaline pH Leads to Increased Metastatic Potential of Estrogen Receptor Silenced Endocrine Resistant Breast Cancer Cells

**DOI:** 10.1371/journal.pone.0076327

**Published:** 2013-10-01

**Authors:** Maitham A. Khajah, Iman Almohri, Princy M. Mathew, Yunus A. Luqmani

**Affiliations:** Faculty of Pharmacy, Kuwait University, Safat, Kuwait; China Medical University, Taiwan

## Abstract

**Introduction:**

Endocrine resistance in breast cancer is associated with enhanced metastatic potential and poor clinical outcome, presenting a significant therapeutic challenge. We have established several endocrine insensitive breast cancer lines by shRNA induced depletion of estrogen receptor (ER) by transfection of MCF-7 cells which all exhibit enhanced expression profile of mesenchymal markers with reduction of epithelial markers, indicating an epithelial to mesenchymal transition. In this study we describe their behaviour in response to change in extracellular pH, an important factor controlling cell motility and metastasis.

**Methods:**

Morphological changes associated with cell exposure to extracellular alkaline pH were assessed by live cell microscopy and the effect of various ion pumps on this behavior was investigated by pretreatment with chemical inhibitors. The activity and expression profile of key signaling molecules was assessed by western blotting. Cell motility and invasion were examined by scratch and under-agarose assays respectively. Total matrix metalloproteinase (MMP) activity and specifically of MMP2/9 was assessed in conditioned medium in response to brief alkaline pH exposure.

**Results:**

Exposure of ER –ve but not ER +ve breast cancer cells to extracellular alkaline pH resulted in cell shrinkage and spherical appearance (termed *contractolation*); this was reversed by returning the pH back to 7.4. Contractolation was blocked by targeting the Na^+^/K^+^ and Na^+^/H^+^ pumps with specific chemical inhibitors. The activity and expression profile of key signaling molecules critical for cell adhesion were modulated by the exposure to alkaline pH. Brief exposure to alkaline pH enhanced MMP2/9 activity and the invasive potential of ER –ve cells in response to serum components and epithelial growth factor stimulation without affecting unhindered motility.

**Conclusions:**

Endocrine resistant breast cancer cells behave very differently to estrogen responsive cells in alkaline pH, with enhanced invasive potential; these studies emphasise the crucial influence of extracellular pH and caution against indiscriminate application of alkalinising drug therapy.

## Introduction

Endocrine resistance, due both to *de novo* and particularly *acquired* refractiveness following exposure to anti–estrogens [1], presents significant challenges for breast cancer therapy that result in increased invasiveness and metastasis, and poor clinical prognosis. Many potential mechanisms have been proposed [2,3] through the establishment of a number of *in vitro* models mostly generated through either adaptation of breast cancer cells to long term estrogen deprivation [4], or by cell survival in the presence of low levels of tamoxifen [5,6,7]. We have previously described several endocrine insensitive cell lines generated by shRNA induced depletion of estrogen receptor (ER) by transfection of MCF-7 cells [8,9]. These lines exhibit distinct changes in morphology, reduced expression profile of epithelial markers such as E-cadherin, catenin, occludins, and claudins, enhanced expression of mesenchymal-associated markers such as N-cadherin, vimentin, integrin β4 and α5 and various metalloproteinase (MMPs), and enhanced motility and invasive potential compared to the parental cells. This is indicative of an epithelial to mesenchymal transition (EMT) [8,10], a process that is now being increasingly implicated in facilitation of breast cancer metastasis. Several markers that are up-regulated during EMT are positively correlated with enhanced invasion and poor prognosis [11,12]. Epithelial cells generally exhibit highly polarized morphology forming extensive junctional complexes and an elaborate cytoskeletal network. The loss of cell adhesion molecules, particularly E-cadherin that is an integral component of adherens junctions, is a disruptive process that allows cellular disaggregation, loss of baso-lateral orientation and dispersion- a feature characterizing mesenchymal cells – and also displayed in all our ER–silenced cells. Several signaling pathways have been implicated in EMT that involve a switch from an essentially keratin based network to one involving vimentin partly through nuclear factor ƙB which also promotes activation of N-cadherin through the basic-helix-loop-helix transcription factor Twist [13]. Other key downstream modifiers of intracellular activity such as Snail, Slug and Sip-1, and the TGF β mediated Smad-dependent pathways all contribute to mesenchymal-like behaviour and have been extensively described [1,14].

It is generally accepted that the tumor microenvironment plays a critical role in the development and progression of the tumor through enhancement of various signaling pathways regulating EMT, cell motility and invasion. In normal cells, the intracellular pH is generally considered to be lower than that in the extracellular space. However, cancer cells have a higher intracellular pH and a lower (acidic) extracellular pH [15,16,17]. It is proposed that this reversed pH gradient serves to enhance cell invasion [18] and increase cancer cell metastasis through various mechanisms that include enhanced CDC42 activity [19,20], assembly of actin filaments [21,22,23,24,25,26,27], osmotic swelling [28], invadopodia formation and maturation [17,29], and up-regulation of the activity of various MMPs [30,31,32].

In this study, we report that alkalinisation (pH 7.7-8.3) of the extracellular environment induces marked morphological changes in ER –ve but not in ER +ve breast cancer cell lines; individual cells rapidly appear to shrink and become spherical, showing a general tendency to disaggregate from the cluster of cells. We demonstrate a modified level of expression and activity of various signaling molecules, enhanced MMP2/9 activity, and enhanced invasive potential toward serum components and EGF in response to increased extracellular pH. All of these morphological and functional changes could be inhibited by various drugs which target two main ion pumps; Na^+^/K^+^ and the Na^+^/H^+^ exchangers. These observations may have important implications not only in relation to drug therapy but also for understanding the mechanisms responsible for tumour metastasis in endocrine resistant cells.

## Materials and Methods

### Cell lines

HBL100 normal breast epithelial cell line, MCF-7 and MDA-MB-231 human breast carcinoma cell lines and PC3 prostate cancer cell line were obtained from the ATCC (American Type Culture Collection, VA, USA). pII, IM-25, IM-26, YS2.5 and YS1.2 cell lines were established in this laboratory by transfection of MCF-7 with ER directed shRNA plasmid as described previously [8,9,10]. Human Embryonic Kidney (HEK293) and HEK499 transfected with RKIP targeting shRNA, were kindly supplied by F. Al-Mulla (Kuwait University). For routine culture all cell lines were maintained as monolayers at 37°C in an incubator gassed with an atmosphere of 5% CO_2_ at 95% humidity, in advanced dulbecco’s minimum essential medium (DMEM) containing phenol red as a pH indicator and supplemented with 5% fetal bovine serum (FBS), 600 µg/ml L-glutamine, 100 U/ml penicillin, 100 µg/ml streptomycin and 6 ml/500 ml 100 x non-essential amino acids (all from Invitrogen, CA, USA) (complete medium). This medium requires an atmosphere of 5% CO_2_ to produce HCO_3_ buffering capacity to maintain pH at 7.4 for normal cell growth. For YS2.5 and YS1.2, the maintenance medium also contained G418 (1mg/ml) but this was omitted during experiments. It should also be noted that upon exposure to normal atmosphere, this medium reached a maximum pH value of approximately 8.2-8.3 (irrespective of the presence of cells). Unless otherwise specified, the term alkaline conditions in the text refers to this pH.

### Microscopic analysis of morphological changes in response to pH

For each cell line, approximately 10^5^ cells were seeded into wells of a 12-well plate and allowed to settle at 37°C for 24 h. Plates were then removed from the incubator (i.e. from the 5% CO_2_ atmosphere needed to maintain the buffering capacity of the DMEM) and exposed to the normal atmospheric environment. Except for the initial experiments where plates were left on the outside bench, all reference to normal atmosphere should be taken to mean placement of cells in an ungassed incubator at 37 °C. Returning cells to 5% CO2 conditions (and hence to pH 7.4) after exposure to normal atmosphere is termed ‘recovery’. Several fields containing colonies were marked and photographed immediately using an Olympus inverted microscope fitted with a camera. Further photographic images of the same marked areas were then recorded periodically over the next 90 min. During the first 10-20 min the medium changed color from orange-red to purple, indicating a rise in pH from 7.4 to about 8.3 (as separately ascertained using a pH electrode). It should be noted that the rate of pH change of the medium was dependent upon the vessel used and the volume of the medium; it was independent of the presence of cells, being governed only by the time taken for the atmosphere to change. Resultant changes in cell size and shape (termed contractolation thereafter) in each photographed field were quantified using Adobe Photoshop CS4 Measuring Tool in terms of the field area occupied by cells.

Other similar experiments were performed with cells growing (for at least 48h) in multiwall dishes that had been pre-coated with either collagen or fibronectin.

### Effect of ion channel protein inhibitors

The following inhibitors (all purchased from Sigma, USA) were used to examine their effect on pH induced cell contractolation: Amiloride (Na^+^H^+^ transport inhibitor), Zoniporide (Na^+^/H^+^ exchanger isoform I [NHE-1] inhibitor), Ouabain and 3, 4, 5, 6-tetrahydroxyxanthone (Na^+^/K^+^ ATPase inhibitors), Nigericin (exchanges K^+^ for H^+^ across biological membranes through the potassium inophone), Bafilomycin A1 (vacuolar type H+ ATPase [V-ATPase] inhibitor), and Phloretin (glucose uptake inhibitor). Stock solutions (10mM) were prepared in dimethyl sulfoxide (DMSO) and stored in small aliquots at -20°C and diluted in DMEM media just prior to performing the experiments. About 10^5^ cells were seeded into a 12-well plate and incubated at 37°C with 5% CO_2_. After 24 h cells were pre-treated for 1 h with various concentrations of each inhibitor (or carrier only; termed no drug) and then exposed to atmospheric conditions to effect the pH change. Cells were microscopically examined and quantification performed as described above.

### Live cell microscopy

The general growth characteristics of cells was continuously monitored by time-lapse photography using a live cell imager (Cell Observer HS, Zeiss, Germany). Cell monolayers grown overnight in an atmosphere of 5% CO_2_ inside a 25 cm^2^ tissue culture flask containing 4 ml DMEM were placed inside the imaging chamber which was also maintained at 37°C with 5% CO_2_ atmosphere. Flasks were positioned to enable photography of cell islands composed of 3-4 cells with images being recorded at 20X magnification every 5 min over a 72 h period. The AxioVision software (Zeiss) was used to combine all the pictures to generate a video of 14 h which was then speeded up to a few minutes using Windows Movie Maker software (Microsoft). For pH induced effects, cells were observed for shorter periods in petri dishes left under normal atmospheric conditions or, for recovery, back in the 5% CO_2_ atmosphere.

### Western blotting

The level of total and phosphorylated ERK1/2, p38 MAPK and Akt, junctional adhesion molecule (JAM-1 and 2), integrin α2, focal adhesion kinase (FAK), heat shock factor (HSF-1), heat shock proteins (HSP-40, 60, 70, and 90), BIP and actin protein was determined in cell lines by immunoblotting (all antibodies were obtained from Cell Signaling, USA). Cells were cultured in 6 well plates with complete DMEM to 80-90% confluence. Cells were treated in one of three ways: 1), harvested immediately after removing the plate from the 37°C, 5% CO_2_ incubator (pH= 7.4) 2), exposed to external atmosphere for 5-60 min (contractolation, pH 7.7-8.3) or 3), exposed to external atmosphere for 60 min (pH changed to 8.3) then returned back to the 37°C, 5% CO_2_ incubator for 60-120 min (pH back to 7.4). In each case, medium was subsequently aspirated off and cell monolayers were harvested by scraping and re-suspension into 300µl of lysis buffer containing 50mM HEPES, 50mM NaCl, 5mM EDTA 1% Triton X, 100µg/ml PMSF, 10µg/ml aprotinin, and 10µg/ml leupeptin. Protein concentration was determined by the Bradford assay using BSA as standard, and 8 µg protein lysate was mixed with an equal volume of 2 x SDS and heated at 90°C for 10 min. Samples were loaded onto a 10% SDS-polyacrylamide gel and electrophoresed at 150 V for 1 h. Proteins were transferred to a nitrocellulose membrane and blocked with 2% BSA for 1 h before being incubated overnight at 4°C with pERK1/2 or pp38 MAPK antibody (1/1000 dilution), total or pAkt antibody (Ser473) (1/1000 dilution), total JAM1 and 2, integrin α2, FAK, BIP, HSP (40, 60, 70, and 90), or HSF1 antibody (1/500 dilution), and actin antibody (1/1000 dilution) prepared in 2% BSA. The membrane was washed and incubated with anti-HRP-conjugated secondary antibody (Cell Signaling) (1/500 dilution) for 1 h, developed with Super Signal ECL and visualized with Kodak X-ray film.

### Motility (scratch) assay

To determine the effect of alkaline pH on cell motility, pII cells were cultured in 6 well plates with complete DMEM to 80-90% confluence. A scratch was created in the cell monolayer using a sterile p1000 pipette tip and a photograph of the scratched area was taken immediately (0 h). The plates were then either placed immediately in the 37°C, 5% CO_2_ incubator or exposed to external atmosphere for 1h (to effect pH change) then placed back in the 37°C, 5% CO_2_. After overnight incubation, another photograph was taken of the same scratched area. The width of the scratch at 24h was calculated as a percentage of the width at 0 h ; a minimum of 3 three areas along the scratch were measured.

### Cell invasion assay

Ultra-pure agarose (Invitrogen) was melted in PBS and once cooled below 40°C, supplemented with complete DMEM and allowed to solidify in individual wells of 6 well dishes at room temperature. Once set, 1-2 sample chambers (3.5 mm in diameter) were created in the gel, 2.5 mm apart in a horizontal line, by insertion of a metallic mould as described previously [10]. Exponentially growing cells (4x10^4^) were re-suspended in complete DMEM and loaded into formed chambers as appropriate for the experiment. Plates were placed either at a), 37°C in the 5% CO_2_ incubator overnight (pH 7.4) b), at 37°C in un-gassed incubator for 1-2 h to induce cell contractolation then placed into the 5% CO_2_ incubator overnight (contractolated 1-2 h) or c), placed at 37°C overnight in un-gassed incubator (contractolated for 24 h). After 24 h, cells that had penetrated into the agarose were manually counted by visual microscopic examination. Random cell invasion was determined as the total number of cells which moved in both lateral directions from the well.

When the effect of EGF was being studied, the agarose was mixed with DMEM containing insulin-transferrin-selenium (ITS; from Invitrogen) in place of the FBS. EGF (Sigma) stock solutions were prepared in sterile PBS and stored in small aliquots at -20°C and diluted in sterile PBS just prior to performing experiments. Random cell invasion in response to EGF stimulation was determined in cells exposed to pH 7.4 overnight or exposed to alkaline pH for 1h (contractolated 1h) then placed back in the gassed incubator overnight.

In another experimental set-up, the effect of amiloride and zoniporide (10µM) on pII cell invasion was determined. Cells were either un-treated or treated with the inhibitors for 1 h then either placed overnight at 37°C in a 5% CO_2_ incubator (pH 7.4), or left for 1 h in an un-gassed incubator and then transferred to the 5% CO_2_ incubator overnight (contractolated for 1 h).

### General matrix metalloproteinase (MMP) activity assay

The general activity of MMP enzymes was determined using an assay kit from Abcam, UK (ab112146) according to the manufacturer’s protocol. In brief, pII or YS1.2 cells (10^4^) were seeded into triplicate wells of 6-well plates and allowed to attach overnight, then starved with serum free media for another 18 h. Control (pH 7.4) or contractolated (for 1 h) cells were then treated with vehicle, or EGF (50 ng/ml) for 30 min and the MMP activity was assayed in the conditioned media; for this, 25 µl of medium was removed and added to 25 µl of 2 mM APMA working solution and incubated for 15 min at 20°C followed by addition of 50 µl of the green substrate solution supplied in the kit - a broad spectrum MMP fluorogenic peptide substrate. After 1h, fluorescence in the sample wells was measured using a microplate reader with a filter set for excitation/emission of 490/525 nm.

### MMP-2/MMP-9 activity assay

The activity of MMP-2 and MMP-9 enzymes was determined using an assay kit from Calbiochem (CBA003) according to the manufacturer’s protocol. Cells were maintained/treated as previously described for the general MMP activity assay. The MMP-2 and MMP-9 activity was assayed in the conditioned media; for this, 90 µl of medium was removed and added to 10 µl of substrate working solution and incubated for 18 h at 37°C, in 5% CO_2_ incubator. A measurement was then performed using a microplate reader with a filter set for excitation/emission of 320/405 nm.

## Statistical Analysis

Student’s two tailed unpaired t-test was used to compare means of individual groups: p ≤ 0.05 was considered statistically significant.

## Results

### Effect of pH on morphological transformation of cells in monolayer culture

**Figure 1:**
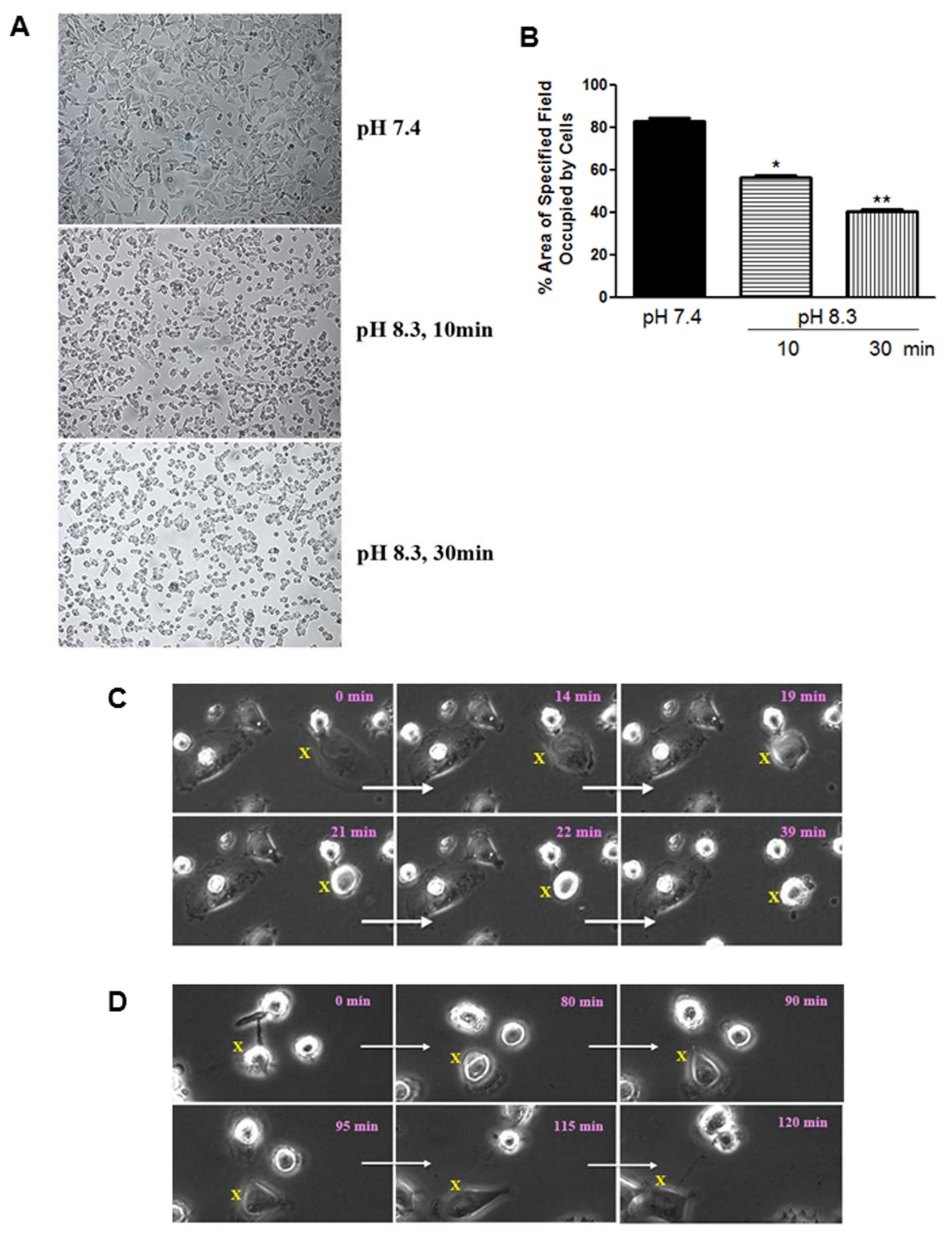
Effect of pH on contractolation of IM-26 cells in monolayer culture. (*A*) Cells grown in complete medium in a 12 well plate were photographed immediately after removal from the CO_2_ incubator (pH 7.4) and after exposure to atmospheric conditions for 10 and 30 min (as the pH increased to 8.3). (*B*) Area occupied by the cells as quantified with Adobe Photoshop CS4 Measuring Tool. *Histobars represent* means ± SEM of at least 3 separate fields of at least 5 independent determinations. Asterisks denote significant difference from the pH 7.4 with *p = 0.01, and **p = 0.005. (*C*) Snapshots from time lapse photography of IM-26 cells undergoing pH-induced contractolation. The initial cell (labeled ‘X’) changes completely over 39 min and moves away from its neighboring cell. (*D*) Snapshots for cells changing back to normal shape after pH change back from 8.3 to 7.4 (recovery). Elapsed time in min is indicated in each snapshot. 20 x magnification.

The upper panel in Figure 1A shows IM-26 cells photographed immediately after removing the microtitre plate from the incubator when the culture medium was at pH 7.4 while the lower panel shows the same cells left on the bench after 10 and 30 min at which time the pH of the medium had become noticeably alkaline within the first 5-10 min - as indicated by the purple color of the phenol red. A dramatic morphological change was observed where cells separated from each other and appeared to shrink in size (we have termed this phenomenon ‘*contractolation*’). This effect was also seen if cells were left in an un-gassed incubator at 37°C confirming the effect to be pH and not temperature dependent. Upon returning the plate to the CO_2_ gassed incubator the pH of the medium was restored to 7.4 within 15-20 min and the cells returned to their original morphology and distribution, though this usually took 2-3 h, and grew normally thereafter. The effect could be quantified, showing a significantly reduced total surface area occupied by transiting cells in a given microscope field (Figure 1B).

IM-26 cells cultured in small petri dishes (which allows for the rapid pH change) were also observed by live cell imaging microscopy. Figure 1C shows snapshot images taken over 30 min during exposure to normal atmosphere; following a random cell marked with X clearly illustrates the pH induced change. In another experiment, cells that had been at alkaline pH for about 30 min were then exposed to a 5% CO_2_ atmosphere in the observation chamber to restore the pH to 7.4. The snapshots in Figure 1D show the reversion of cells to their original appearance after about 2 h; clearly exemplified by following the cell marked X. Movie S1 and Movie S2 contains the time lapse video images which show the progressive change described in Figure 1 C and D respectively.

**Figure 2:**
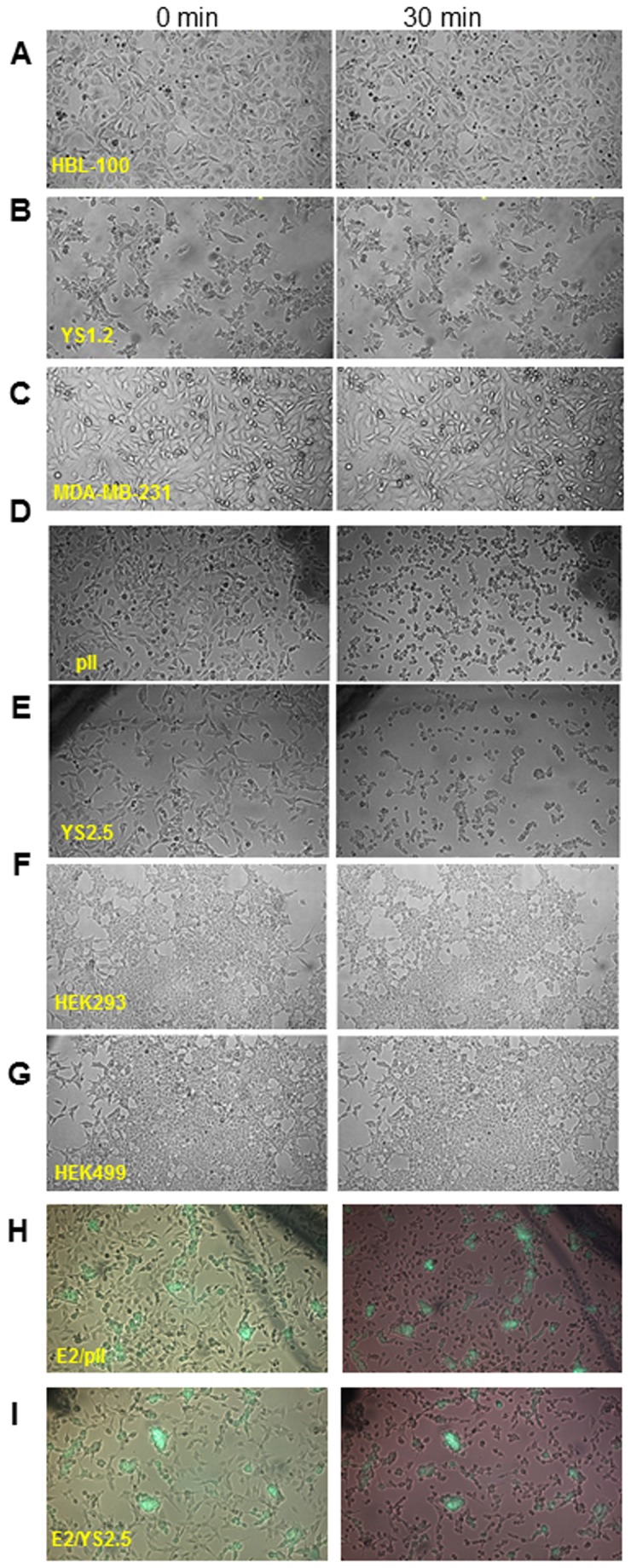
Effect of pH on contractolation of breast and non-breast cell lines. Cells were photographed immediately after removal from the CO_2_ incubator (0 min, pH 7.4) and after 30 min of exposure to normal atmospheric conditions (as the pH increased to 8.3). The following cell lines are shown: (*A*) HBL-100, (*B*) YS1.2, (*C*) MDA-MB-231, (*D*) pII, (*E*) YS2.5, (*F*) HEK 293, and (*G*) HEK 499. Panels (*H*) and (*I*) represent the combined phase contrast and fluorescence micrographs for the co-culture of EII cells (which express a transfected green fluorescent protein) with either pII or YS2.5 cells respectively.

The same experiment was subsequently conducted on other cell lines. In contrast to the observations on IM-26, no gross morphological change was observed even after periods of 60-90 min at pH 8.3 in the case of the parental MCF-7 ER + cells, the shRNA transfected but ER+ YS1.2 and EII lines or the normal breast cell line HBL100 (Figure 2); all retained their usual appearance seen at pH 7.4. The ER –ve MDA-MB-231 cell line showed some indication of change but only after about 2h at the alkaline pH. Three other cell lines which exhibit shRNA induced ER silencing (IM-25, pII and YS2.5) all behaved identically to the IM-26. Prolonged periods of alkalinity beyond 3h resulted in irreversible cell death. Several non-breast lines were also examined: immortalized human embryonic kidney (HEK293) cells and a derivative line (HEK499) established from the parent by transfection with miRNA vector designed to silence the RKIP gene, and a prostate cancer line (PC3); none showed the pH induced contractolation.

In a co-culture experiment, EII cells (which express a transfected green fluorescent protein) were grown with either pII or YS2.5 cells and exposed to the alkaline pH. The combined phase contrast and fluorescence micrographs shown in Figure 2 H and I, illustrate the differential response; both pII and YS2.5 contractolated whereas the (fluorescent) EII cells remained unchanged.

It should be noted that the morphological changes observed in response to alkaline pH are remarkably similar to changes observed during cell division where cells shrink and become round prior to mitosis, confirming that this transformation is neither artifactual nor damaging (Movie S3 and Movie S4 contains the time lapse video images which show cell division in MCF-7 and IM-26 respectively). Of course the time course for the two processes is completely different with contractolation occurring in a few minutes while cell division occurs at approximately 12-18h in these cells.

### Effect of substratum on pH-induced contractolation

**Figure 3:**
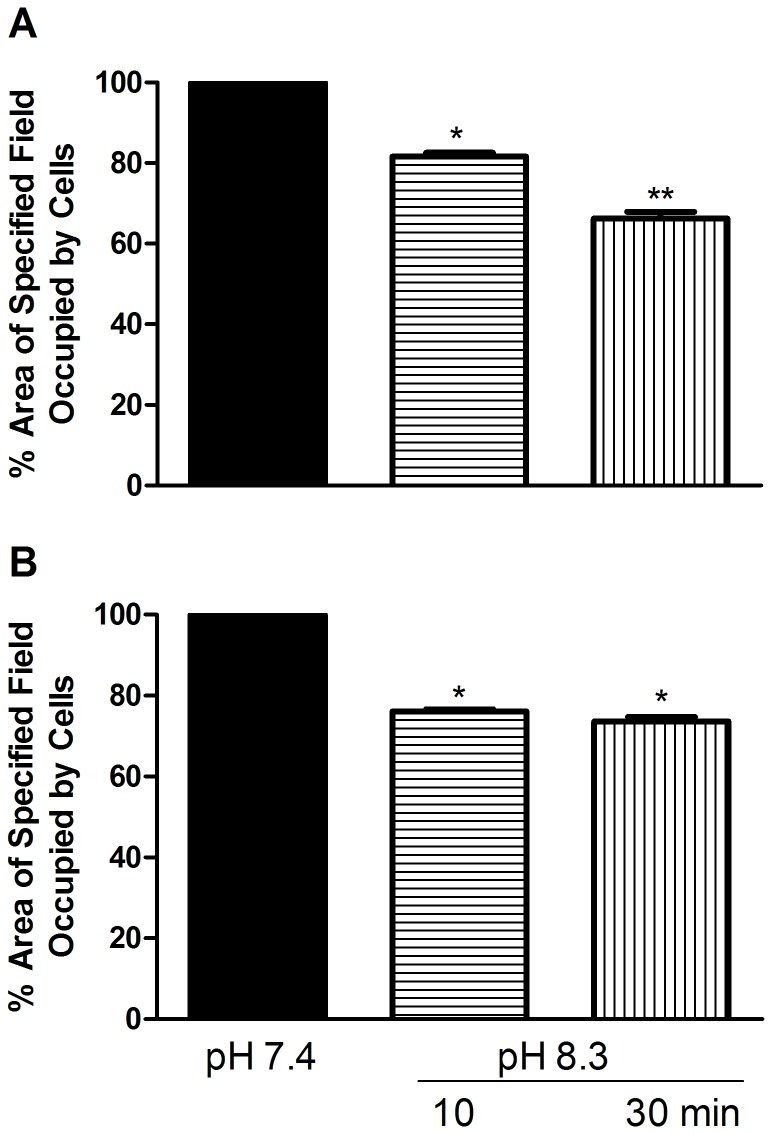
Effect of substratum on pH-induced contractolation of IM-26 cells. Cells were seeded into 24-well microwell plates coated with either fibronectin or collagen, left to grow for 48h in a 5% CO_2_ atmosphere (pH 7.4; solid bars) and then exposed to normal atmospheric conditions for 10 and 30 min (as pH increased to 8.3; hatched bars). Panel A and B show quantitative analysis of at least 3 separate fields measured with Adobe Photoshop CS4 Measuring Tool (means ± SEM) for cells coated with fibronectin and collagen respectively. Asterisks denote significant difference from pH 7.4 (set as 100%) with *p = 0.03, and **p = 0.02.

In order to determine whether the contractolation effect is dependent upon the surface to which the cells are attached, cultures were grown in microwell plates coated with either fibronectin, or collagen. Five different lines were used for this experiment (MCF-7, YS1.2, HBL100, MDA-MB-231, and IM-26) and seeded at the same density onto the two different surfaces. All the cell lines grew normally on collagen and fibronectin coated plates. Exposure to the alkaline conditions had no effect on any of the cell lines tested except IM-26 in which the contractolation phenomenon was observed after 15 min (during pH change) on both collagen and fibronectin. Quantitative analysis of several microscope fields showed about 20% reduction in the area occupied by the IM-26 cells after contractolation on both fibronectin and collagen (Figure 3).

### Effect of ion pump inhibitors on contractolation

**Figure 4:**
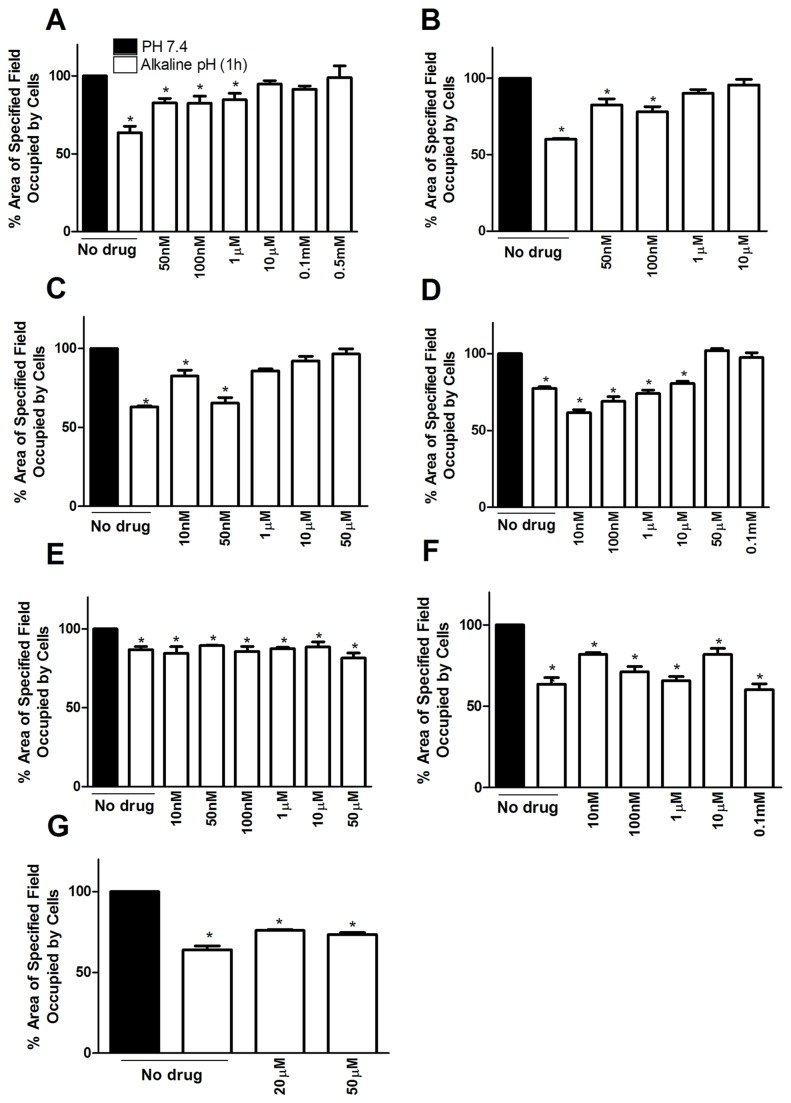
Effect of ion pump inhibitors on contractolation. Quantitative analysis of pH induced contractolation of pII cells exposed to pH 7.4 (solid bars) or after exposure to alkaline pH for 1h (open bars). The following inhibitors were used: (A) amiloride, (B) zoniporide, (C) ouabain, (D) 3, 4,5, 6-tetrahydroxyxanthone, (E) nigericin, (F) bafilomycin A1 and (G) phloretin. Histobars represent means ± SEM of at least 3 independent determinations. Asterisk denotes significant difference from the pH 7.4 condition (set as 100%) with *p ≤ 0.05.

Figure 4 shows the effect of several drugs known to affect ion channels. Amiloride has been reported in the literature as an inhibitor of the Na^+^/H^+^ antiport across plasma membranes, which can exert an important influence on the pH environment. It completely prevented contractolation of pII cells at concentrations of 10µM upwards (panel A). Similarly, zoniporide, a more selective inhibitor of the Na^+^/H^+^ exchanger isoform 1 (NHE1) significantly inhibited contractolation at 1µM (panel B). Two inhibitors of the Na^+^/K^+^ ATPase pump, ouabain and 3, 4, 5, 6-tetrahydroxyxanthone, also abolished contractolation at 1µM and 50µM respectively (panels C, D). Nigericin, which dissipates proton gradients by exchanging K^+^ for H^+^ across biological membranes through the potassium inophore, had no effect at doses between 10nM-50µM (panel E), and at higher concentrations, caused rapid cell detachment. We also used bafilomycin A1 to inhibit the vacuolar type H^+^ ATPase (V-ATPase) at similar dose ranges used with the other drugs but it had no effect on the pH induced contractolation (panel F). To determine whether contractolation might be energy dependent, we tested the effect of phloretin, a dihydrochalcone that inhibits Na^+^ dependent carrier-mediated uptake of D-glucose which is required by cancer cells as a major energy source. It was found to have no effect (panel G). All of these drugs were subsequently tested on the other ER silenced cell lines IM-26 and YS2.5 with very similar results (data not shown).

### Association of pH induced contractolation with activation of intracellular signaling molecules

**Figure 5:**
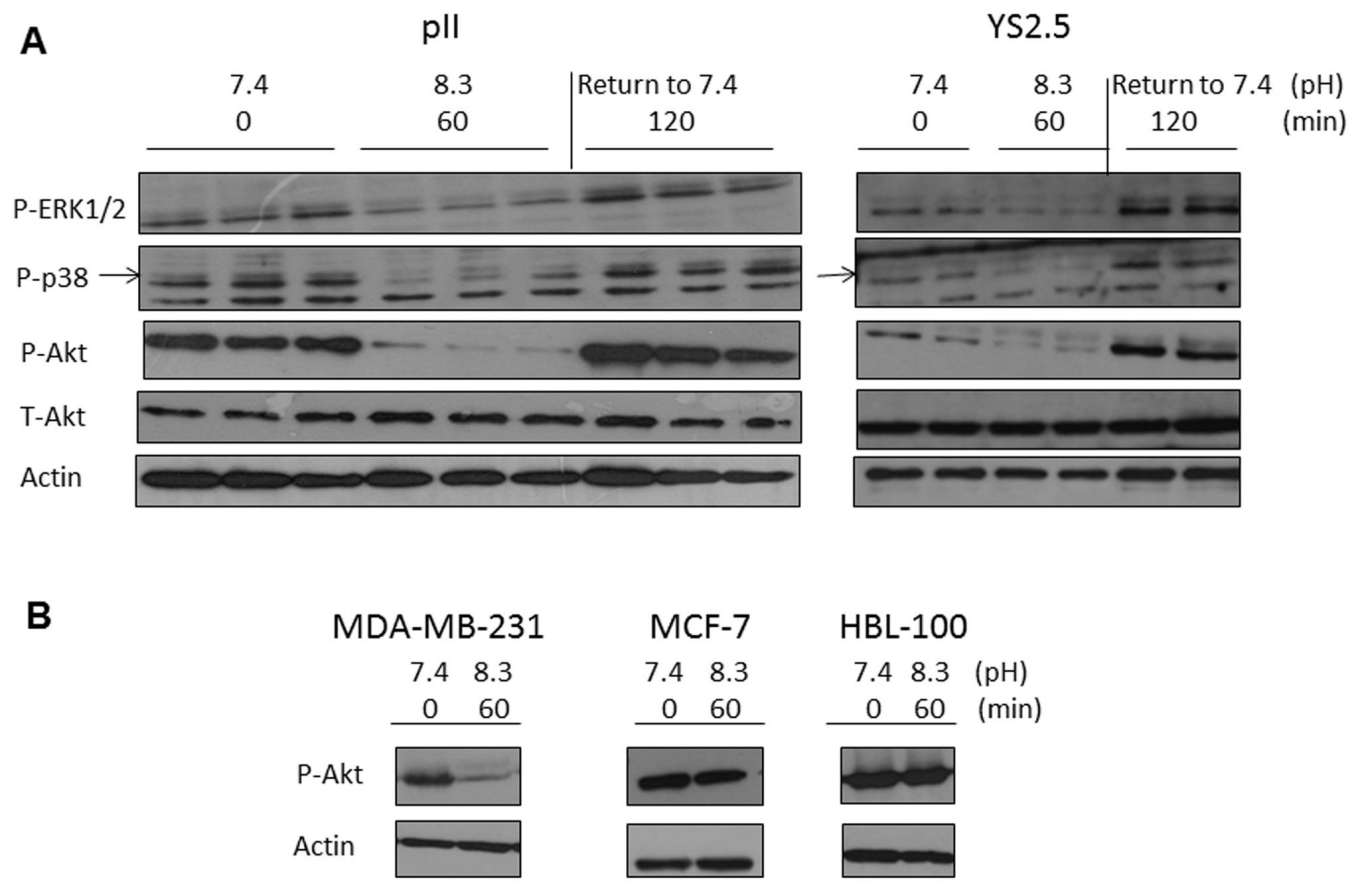
Association of pH induced contractolation with activation of intracellular signaling molecules. Cells were lysed immediately after removal from the incubator (0 min, pH 7.4), after 60 min exposure to normal atmosphere (as the pH increased to 8.3), or after 60 min exposure to normal atmosphere and returning back to pH 7.4 for 120 min (recovery). The pH of the medium was measured at the time of cell lysis and is indicated under each test condition. (A) pII and YS2.5 and (B) MDA-MB-231, MCF-7 and HBL-100 cell lysate proteins (8 µg) were electrophoresed on 10% SDS polyacrylamide gel, blotted onto nitrocellulose membrane and probed with antisera to p-ERK 1/2, p-p38 MAPK, p-Akt, total-Akt and actin as described in Methods.

As pH is likely to affect the function of membrane associated molecules, we examined the activation of several key regulators of signal transduction by comparing their levels in protein extracts of cells that had been i), maintained at pH 7.4 ii), exposed to alkaline pH for 60 min and iii), returned to pH 7.4 for about 2h prior to harvesting, after alkaline exposure. Panel A in Figure 5 shows the total and phosphorylated form of Akt and of pERK1/2 and pp38 MAPK in pII and YS2.5 cells. Cells exposed to alkaline pH showed marked reduction in pAkt with no change in level of total Akt; similarly there were parallel reductions in pERK1/2 and pp38 MAPK. Upon transferring the cells back to their normal pH conditions of 7.4, the phosphorylated forms of all three proteins returned to, or even exceeded their original amounts. The ER-ve MDA-MB-231 cells also showed a pH induced fall in pAkt whereas no change was observed for either MCF-7 or the normal breast cell line HBL100 (Panel B in Figure 5).

**Figure 6:**
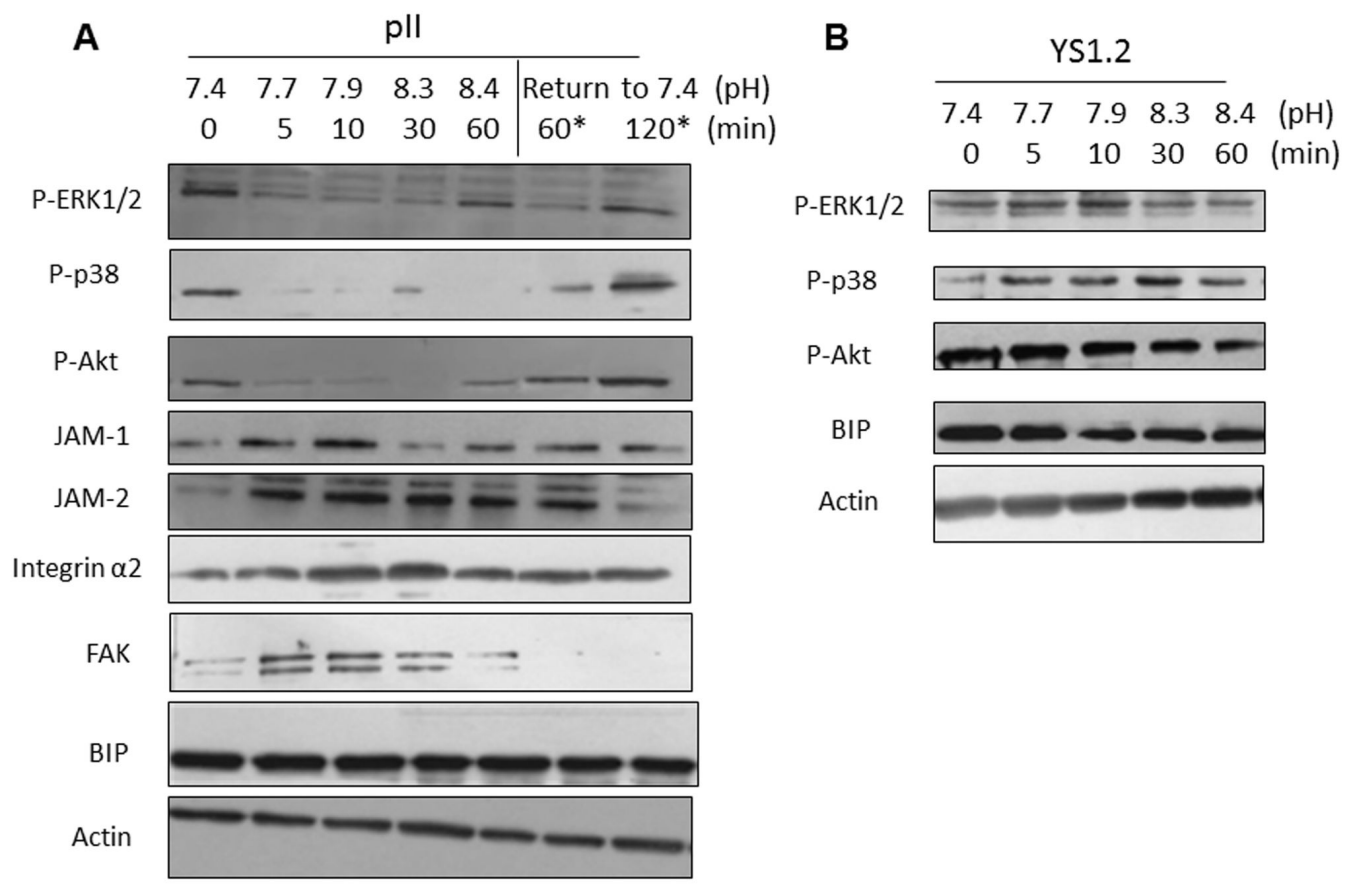
Temporal association of pH induced contractolation with activation of intracellular signaling molecules and other proteins necessary for cell morphology/function. Cells were lysed immediately after removal from the CO_2_ incubator (0 min, pH 7.4), after various time points of exposure to normal atmosphere (5-60 min), or after 60 min exposure to normal atmosphere and returning back to pH 7.4 for 60 or 120 min (recovery). The pH of the medium was measured at the time of cell lysis and is indicated under each test condition. (A) pII and (B) YS1.2 cell lysate proteins (8 µg) were electrophoresed on 10% SDS polyacrylamide gel, blotted onto nitrocellulose membrane and probed with antisera to p-ERK 1/2, p-p38 MAPK, p-Akt, JAM-1 and -2, integrin α2, FAK, BIP and actin as described in Methods.

In order to determine more precisely the relationship between extracellular pH and expression of signaling molecules, we harvested pII cells that had been exposed to atmospheric conditions for various times up to 60 min (simultaneously measuring the pH of the medium at each point) and then after ‘recovery’ for a further 2h. The western blot depicted in Panel A Figure 6 shows a reduction in the level of pERK1/2, pp38, and pAkt as early as 5 min (when pH reached 7.7) and which remained low for the subsequent period as pH increased to a maximum value of 8.4, returning to original levels after 60-120 min of ‘recovery’. A similar experiment was performed with YS1.2 cells (shRNA transfected but not ER down-regulated) and no change was seen at any pH (Panel B in Figure 6). We also measured the levels of several other molecules that have been reported to influence cell morphology and function. JAM-2 levels were immediately increased from 5 min onwards, returning back to original levels after 2h ‘recovery’. JAM-1and FAK peaked at 5 and 10 min (pH 7.7-7.9) whilst integrin α2 was maximal at 10 and 30 min (pH 7.9-8.3) and all declined thereafter. There was no change in β actin or in what we took to be an irrelevant molecule to this phenomenon; BIP.

**Figure 7:**
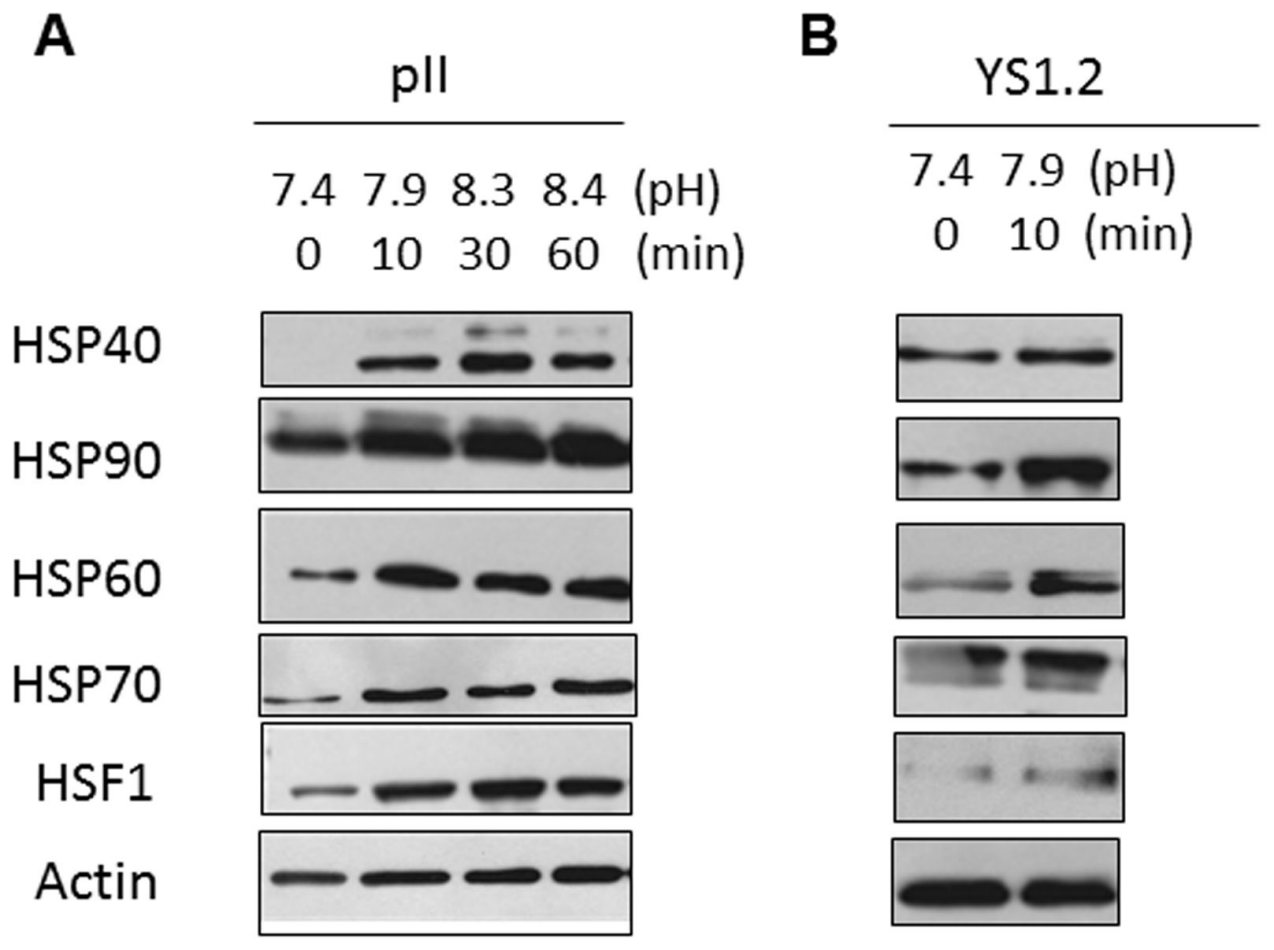
Association of pH induced contractolation with expression of heat shock proteins. Cells were lysed immediately after removal from the CO_2_ incubator (0 min, pH 7.4),or after 10-60 min exposure to normal atmosphere. The pH of the medium was measured at the time of cell lysis and is indicated under each test condition. (A) pII and (B) YS1.2 cell lysate proteins (8 µg) were electrophoresed on 10% SDS polyacrylamide gel, blotted onto nitrocellulose membrane and probed with antisera to HSPs 40, 90, 60, 70, HSF1 and actin as described in Methods.

Exposure to alkaline pH may understandably be regarded as a form of stress, so we also examined expression of proteins known to be part of the heat shock response. The data in Figure 7A shows that indeed four HSPs as well as HSF1 all increased at all elevated pH conditions in pII cells. Some HSPs; particularly 60 and 90 were also elevated in YS1.2 cells in response to alkaline pH (Figure 7B) although no changes in the activity of signaling molecules was observed (Figure 6B).

### Behavior of contractolated cells in motility and invasion assays

**Figure 8:**
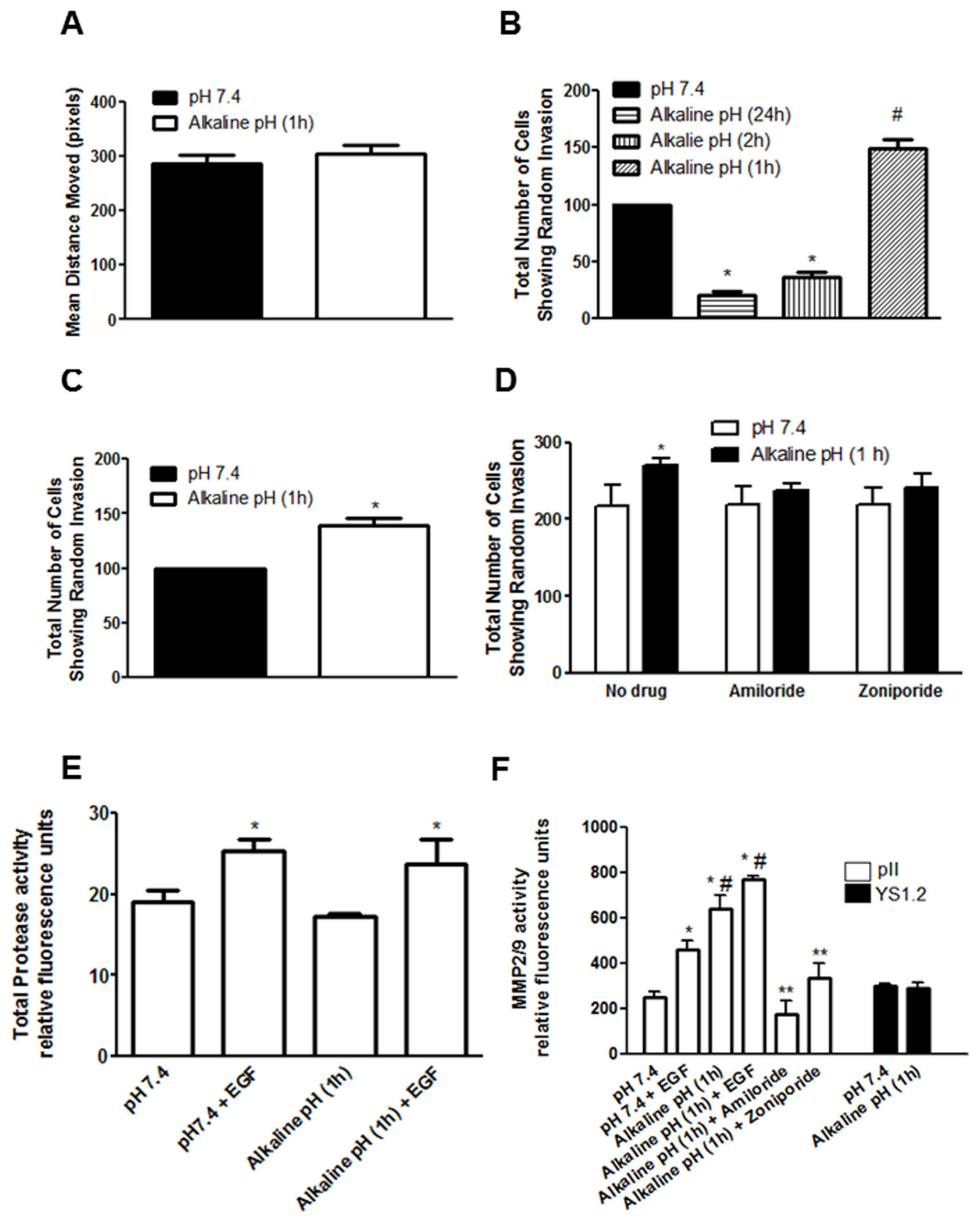
Behavior of contractolated cells in motility and invasion assays, and association with matrix metalloproteinase activity. (A) The mean distance moved (in pixels), as a measurement of cell motility of pII cells after an overnight incubation in either a gassed incubator (pH 7.4, solid bar) or after a brief (1h) culture in un-gassed incubator at 37°C and subsequent overnight incubation in a gassed incubator (open bar). (B) The total number of pII cells showing random invasion (as defined in Methods) after an overnight incubation in a gassed incubator (pH 7.4, solid bar, set as 100%), after culture in un-gassed incubator at 37°C for 1-2h and subsequent overnight incubation in a gassed incubator, or after an overnight incubation in un-gassed incubator at 37°C (hatched bars). Asterisks denote significant difference from pH 7.4 with * p≤0.0001, and # p= 0.0004. (C) The total number of pII cells showing random invasion in response to EGF stimulation (50ng/ml) after either an overnight incubation in a gassed incubator (pH 7.4, set as 100%), or after a brief (1h) culture in un-gassed incubator at 37°C and subsequent overnight incubation in a gassed incubator (open bar). Asterisks denote significant difference from pH 7.4 with * p=0.0024 (D) The effect of amiloride or zoniporide (10µM) treatment of the total number of pII cells showing random invasion after an overnight incubation in a gassed incubator (pH 7.4, open bars), or after a brief (1h) culture in un-gassed incubator at 37°C and subsequent overnight incubation in a gassed incubator (solid bars). Asterisks denote significant difference from pH 7.4 (no drug) with * p=0.01 (E) Effect of pH and EGF stimulation on general MMP activity in pII cells. Cells were either cultured in a gassed incubator (pH 7.4) or in un-gassed incubator for 1h and either left untreated or stimulated with EGF (50ng/ml) for 30 min and metalloproteinase activity was determined using a fluorogenic substrate as described in Methods. Asterisks denote significant difference from no EGF treatment groups with * p≤ 0.05. (F) Effect of pH, EGF, amiloride, and zoniporide treatment of MMP2/9 activity in pII (open bars) and YS1.2 (solid bars) cells. Cells were either cultured in a gassed incubator (pH 7.4) or in un-gassed incubator for 1h and either left untreated, treated with amiloride or zoniporide (10µM, 60 min), or stimulated with EGF (50ng/ml, 30 min) and MMP2/9 activity was determined using a fluorogenic substrate as described in Methods. Asterisks denote significant difference from pH 7.4 with * p≤0.001, from pH7.4 +EGF with # p≤0.0001, and from alkaline pH (1h) with ** p ≤0.001. Bars represent means ± SEM for at least 3 independent determinations.

For the motility assay, pII cells grown for two days to 90% confluency in 6 well plates were either treated as below or after exposure to normal atmosphere for 60 min (maximally contractolated). A scratch was created in the cell monolayer, the width being recorded at several points, and plates left at 37 ^o^C/5% CO_2_ for 24 h followed by a second measurement of the scratch to determine the net movement of cells (see Methods). As shown in Figure 8A, the exposure to alkaline pH did not modify the ability of pII cells to close the wound.

To determine the effect of prior exposure to alkaline pH on their invasive ability, pII cells were first left at atmospheric conditions for different times and then tested in the under-agarose assay. Figure 8B shows that a 1h exposure to alkaline pH significantly enhanced subsequent invasion whereas after 2h it was greatly reduced and after 24h exposure not seen at all (in this condition, no attached cells were seen in the wells and presumed dead). The addition of EGF (which we have previously shown to be the most potent agent in promoting cell invasion [10]) further enhanced random invasion of 1h contractolated cells (Figure 8 C).

Since amiloride and zoniporide treatment inhibited contractolation (Figure 4) we determined their effect on pII cell invasion. Neither agent influenced the *basal* level of invasion but both abolished the *enhanced* invasion seen with (1h) contractolated cells (Figure 8D). Figure S1 illustrates actual cells that have penetrated out of the cell chamber into the surrounding agarose (x10 magnification).

### Association of contractolation with matrix metalloproteinase activity

Total MMP activity in conditioned medium from pII cells was unaffected by a 1h exposure to alkaline pH (Figure 8 E). The addition of EGF increased MMP activity to the same extent under both pH conditions. As MMP2/9 have been more specifically implicated in breast cancer metastasis we also measured these. At pH7.4, EGF increased MMP2/9 activity of pII cells. At alkaline pH both the basal and EGF stimulated activity was greatly enhanced. Amiloride and zoniporide significantly inhibited the pH-induced enhancement to the level seen in cells at pH 7.4. In contrast, MMP2/9 activity in conditioned medium from YS1.2 cells was unaffected by pH change (Figure 8 F).

## Discussion

In previous publications [8,9,10] we have described the expression profile and morphology of several ER silenced breast cancer cell lines that were developed as an *in vitro* model system to study endocrine resistance. A chance observation that the morphology of one of these lines (IM-26 cells) dramatically altered when exposed to alkaline conditions, led us to a systematic examination of this phenomenon. The same behavior was observed for four quite separately established ER –silenced lines, but not for either the parent ER+ MCF7 cells, shRNA transfected lines that failed to down-regulate ER, normal breast epithelial cell line HBL100 or several non-breast cell lines of both normal and cancerous origin. Initially we assumed the cells had scattered, similar to the effect described for hepatocyte growth factor (HGF) treated cells [33,34,35], or by stimulation of c-Met, which in addition to scattering increased proliferation and angiogenesis and enhanced cell motility, invasion and metastasis [36,37]. Also, in breast cancer cells, HGF can induce EMT and enhance cell motility and invasion [38,39,40]. Therefore we tested HGF (1-100 ng/ml) over 1-48 h on both ER +ve and ER silenced cell lines, but we did not find any scattering effect or any enhancement of proliferation or invasion at similar dose ranges used in other studies (data not shown). HGF did however induce scattering of human prostate cancer cell line PC3 (data not shown) at similar dose range used in previous reports [41]. On closer examination, this phenomenon was seen to be quite different from the morphology change of IM-26 and with a much longer time scale (16-24h).

The pH –induced contractolation involves an apparent shrinking of the cell into a more spherical shape with considerable membrane ruffling (to be detailed in a subsequent paper) which is strikingly similar to what is seen in our live cell imaging videos when these (as well as the parent MCF-7) cells are undergoing cell division. In both processes the cells revert completely to their original morphology upon return to pH 7.4, or on completion of mitosis. Thus contractolation is totally reversible and is a physiological response; no deleterious effect of exposure up to 3h to the high pH was apparent on either responding or non-responding cells. But on functional basis, exposure of pII cells to alkaline pH for more than 1h significantly reduced their invasive potential (Figure 8B). That ER-silenced cells lack cell adhesion proteins, critically E-cadherin and catenins with disruption of many junctional component mRNAs [8] and have adopted the more fluid characteristics of mesenchymal cells may facilitate further pH induced separation and detachment from each other and partially from the substratum, whether that be charged plastic or proteinaceous.

Whereas general consensus favors an acidic tumor microenvironment, due in large measure to the extrusion of accumulated lactic acid and protons produced by excessive glycolytic activity [42] to be conducive to metastasis, we provide experimental evidence that 1h exposure to high extracellular pH actually confers increased metastatic potential in *specifically* the endocrine resistant ER-ve breast cancer cells. Interestingly, a similar morphological change was documented for human melanoma cell line MV3 [43] which were dendritic or ameboid in shape at pH 6.6-7 but became spherical at 7.5-8.1. In their case however this shape change *reduced* their invasive potential.

The rapidity of contractolation prompted us to examine activation of intracellular signaling to identify possible mechanisms. Appearance of JAM1 and 2, integrin alpha 2 and FAK, which play a vital role in cell adhesion and migration, was *reversibly* enhanced in alkaline pH. Many components of the extracellular matrix (ECM) modulate functions of cancer cells through both attachment-dependent (focal adhesion-related) or independent mechanisms. For example, interaction of integrin alpha 2-beta 1 receptor complex with collagen type II and laminin coated surfaces results in ligand-induced Smad2 activation [44]. Modification of ECM components modulates the staurosporine-induced apoptotic process of breast cancer cells through modulation in Raf/MAP kinase pathway activity [45]. ECM proteins also influence proliferation and cytokine expression in various human breast cancer cell lines [46]. Rather more unexpectedly, phosphorylation of p38, ERK1/2 and Akt was significantly reduced by exposing pII or YS2.5 (but not MCF-7 or YS1.2) to alkaline pH, with re-activation during recovery phase. The time scale (which in these experiments is related to pH), appears to support the notion that these molecular changes precede the morphological change and could arguably be regarded as cause rather than effect. Given that all of the measured proteins associated with stress response were also rapidly elevated (Figure 7), a plausible scenario may be that alkalinisation initiates a protective shut down in cellular metabolism, reflected in the reduced activity of signaling molecules, and parallel mechanisms lead to contractolation to reduce the cell’s contact with its environment by minimizing its surface area. The failure of endocrine sensitive cells to respond in the same way, and yet still show some signs of stress response, may be due to their inability to dissociate cellular contacts, which might mean that it is the contractolation that triggers slowdown of cell signaling. However since these cells acquire increased invasive propensity, far from entering a dormant state they are directed towards another activity. Returning to their original morphology is associated with re-activation of signaling.

The balance between intra and extracellular pH is regulated through ion channel proteins that include vacuolar H^+^-ATPases (V-ATPases), the Na^+^/H^+^ exchanger (NHE), bicarbonate transporters, monocarboxylate transporters (MCTs) and the carbonic anhydrase enzyme (CA) [18]. The NHE1 isoform regulates various functions of cancer cells by expelling protons from the cytosol to the surrounding environment, contributing to extracellular acidification [47]. There is an enhanced NHE1 expression and activity profile in various forms of cancer [48,49,50,51] and it is mainly localized at the leading edge of migrating cancer cells suggesting a pivotal role in enhancing directional migration and invasion [16,43,52,53,54,55,56]. The other ion pump implicated in cancer cells is the Na^+^/K^+^ ATPase. Its level of expression and functions is also altered in many forms of cancer and it is associated with both cell proliferation [57,58,59,60,61] and adhesion [62,63,64,65,66]. Particularly in the ER -ve MDA-MB-231 cells, ouabain (which inhibits the Na^+^/K^+^ ATPase pump activity) inhibits cell proliferation and modulates the activity of various downstream signaling molecules such as Src, ERK1/2 and JNK [67]. In our study, amiloride and zoniporide which are known to inhibit these ion pumps, were effective in inhibiting contractolation, MMP2/9 activity and invasion. This was unexpected. As these pumps expel H^+^ to the extracellular environment, increasing acidity, their inhibition should *increase contractolation*. Either these agents are affecting some other process or the NHE and ATPase have other functions in non- excitable cells distinct from ion regulation [68,69,70].

Matrix metalloproteinase (MMPs) are zinc-dependent endopeptidases involved in the cleavage of cell surface receptors, the release of apoptotic ligands and chemokine/cytokine inactivation. MMPs are also thought to modify cell proliferation, migration, differentiation, angiogenesis and host defense [71,72]. Particularly in breast cancer, MMP2 and MMP9 have been shown to play a major role in cell migration and invasion and their enhanced levels in patients predicts poor clinical prognosis [73,74,75,76,77]. Interestingly, in a mouse melanoma cell line extracellular acidic pH was reported to induce MMP9 expression through phospholipase D-triggered MAPK activation [78,79]. In our study we found that it was alkaline pH that increased specifically the activity of MMP2/9 in pII (but not in YS1.2) cells (no change was seen in total MMP activity). Furthermore, the higher pH also enhanced the independent stimulatory effect of EGF on specifically MMP 2/9 but not total MMP activity. Amiloride and zoniporide which both inhibited the alkaline enhanced cell invasion also significantly inhibited the enhanced MMP2/9 activity in alkaline medium suggesting a direct, quite likely causal association with contractolation. It should be noted that alkaline pH had no effect on cell motility (Figure 8A) so the enhanced invasion seen might be attributed to enhanced MMP2/9 levels in response to the pH change. It would be instructive to perform a wider proteomic screen to determine the pattern of molecular direction as well its temporal sequence during contractolation and subsequent recovery to determine the underlying molecular mechanisms.

The majority of published data suggests that tumours are surrounded by an acidic extracellular environment as a result of increased ‘aerobic glycolysis’, which encourages cell proliferation, metabolic adaptation and invasion [18,80,81] through various mechanisms such as enhanced activity of CDC42 [19,20], *de novo* assembly of actin filaments and various actin binding proteins [21,22,23,24,25,26,27], osmotic swelling due to enhanced influx of water through aquaprotein channels [28], formation and maturation of invadopodia [17], activation of proteases [17,29], and enhancement of the activity of various MMPs such as MMP 3 and 9 [30,31,32]. From a therapeutic perspective, extracellular pH, and its potential manipulation, can have a significant impact; acidic conditions could favour cellular uptake and efficacy of weakly acidic drugs such as cyclophosphamide and cisplatin, while obstructing those that are weakly basic such as doxorubicin and vinblastine.

It remains to be seen whether the observations presented in this report are a purely *in vitro* phenomenon or how increased metastatic behavior of (alkaline) contractolated cells can be reconciled with contrasted reports that it is tumour environment acidity that, for example, decreases binding of E-cadherin to catenin, leading to cell migration and invasion. The point however, is that it is *only our endocrine resistant cells* which have undergone EMT that display this phenomenon and they already lack these junctional adhesions. If endocrine resistant tumours were to behave similarly *in vivo*, a therapeutic strategy of tumour microenvironment alkalinisation [82] may be counter-productive in such circumstances.

It should also be noted that perhaps, just as intracellular pH is not uniform throughout the cell, there may be localized fluctuations in the pH of the matrix which would not be detected by gross measurements of extracellular pH using microelectrodes or fluorescent dyes. This variation may be created by the modified activity of cells undergoing EMT, for their own advantage. In all likelihood it is these cells that have the greatest propensity for metastasis since endocrine resistant tumours are usually more aggressive.

**Figure 9:**
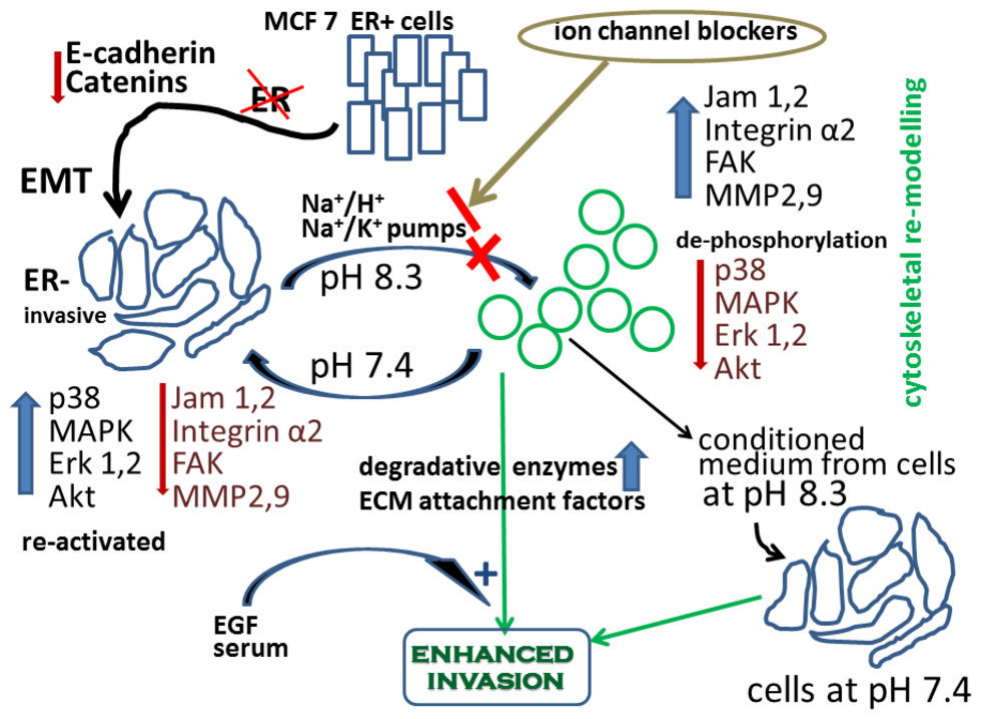
Preliminary model for the contractolation phenomenon. Brief exposure of endocrine resistant ER silenced breast cancer cells (that have undergone EMT) to extracellular alkaline pH environment modulates the activity of Na^+^/H^+^ and Na^+^/K^+^ pumps, resulting in reduced activity of the intracellular signaling molecules p38 MAPK, Akt, and ERK1/2, enhanced expression level of FAK, JAM-1 and 2, and integrin alpha 2, and dramatic morphological changes including cell shrinkage, rounding, and separation from neighboring cells (possibly due to reduced level of E-cadherin and catenins as a result of the EMT process). This leads to enhanced secretion of MMP2/9 in the extracellular environment and enhances cell invasion, particularly that responsive to EGF and serum factors. Conditioned medium from cells at pH 8.3 can confer enhancement of invasion on cells that had been maintained at pH 7.4. The entire contractolation process with accompanying changes can all be reversed by returning cells to pH 7.4.

In Figure 9, we present a preliminary model which describes the contractolation phenomenon. Upon a brief exposure of ER silenced breast cancer cells to extracellular alkaline environment (pH 7.7-8.3), the activity of several ion pumps particularly Na^+^/K^+^ and Na^+^/H^+^ exchangers are modulated which leads to reduced phosphorylation level of three key intracellular signaling molecules; p38 MAPK, Akt, and ERK1/2, and enhanced expression profile of several adhesion molecules such as FAK, JAM-1 and 2, and integrin α 2. This results in marked morphological changes (shrinkage and rounded appearance) possibly due to reduced expression profile of adherent junction proteins (E-cadherin and catenins) in these cells which have undergone the EMT process. The resultant changes lead to enhanced level of MMPs (particularly MMP 2/9) in the extracellular environment and enhanced cell invasion in response to stimulation with serum components and particularly with EGF.

## Conclusions

Numerous reports suggest an acidic microenvironment surrounding tumor cells, which ostensibly aids their dissemination through undefined mechanisms. We present novel data which demonstrates that brief exposure of specifically endocrine resistant breast cancer cells to an alkaline microenvironment enhances their invasive potential, in part through increased MMP2/9 activity which is critical for cell invasion and metastasis, and associated changes in several key signaling molecules. These observations underline the influence that pH can exert on cell adhesion/motility. We caution that newer therapeutic strategies aimed at reversing or even alkalinizing pH of the extracellular matrix may be counter-productive for endocrine resistant cells. Gross measurements of extracellular pH may possibly not be reflective of the immediate microenvironment around cells with high propensity for metastasis.

## Supporting Information

Figure S1 **Cell penetration into the surrounding agarose.** Actual cells that have penetrated out of the cell chamber into the surrounding agarose (x10 magnification) by using the agarose gel assay.
(TIF)

Movie S1 **IM-26 cells undergoing pH-induced contractolation.** Time lapse movie which shows the progressive changes of IM-26 cells undergoing pH-induced contractolation over 39 min.
(WMV)

Movie S2 **IM-26 cells returning back to normal shape after pH-induced contractolation.** Time lapse movie which shows the progressive changes of IM-26 cells back to normal shape after pH change back from 8.3 to 7.4 (recovery).
(WMV)

Movie S3 **MCF-7 cell division.** Time lapse movie which shows the process of MCF-7 cell division.
(WMV)

Movie S4 **IM-26 cell division.** Time lapse movie which shows the process of IM-26 cell division.
(WMV)
